# Pleistocene climate changes shaped the population structure of *Partamona seridoensis* (Apidae, Meliponini), an endemic stingless bee from the Neotropical dry forest

**DOI:** 10.1371/journal.pone.0175725

**Published:** 2017-04-14

**Authors:** Elder Assis Miranda, Kátia Maria Ferreira, Airton Torres Carvalho, Celso Feitosa Martins, Carlo Rivero Fernandes, Marco Antonio Del Lama

**Affiliations:** 1 Laboratório de Genética Evolutiva de Himenópteros, Departamento de Genética e Evolução, Universidade Federal de São Carlos, São Carlos, SP, Brazil; 2 Departamento de Ciências Biológicas, Universidade Estadual de Santa Cruz, Ilhéus, BA, Brazil; 3 Unidade Acadêmica de Serra Talhada, Universidade Federal Rural de Pernambuco, Serra Talhada, PE, Brazil; 4 Departamento de Sistemática e Ecologia, Universidade Federal da Paraíba, João Pessoa, PB, Brazil; 5 União de Ensino Superior de Campina Grande, UNESC, Campina Grande, PB, Brazil; National Cheng Kung University, TAIWAN

## Abstract

*Partamona seridoensis* is an endemic stingless bee from the Caatinga, a Neotropical dry forest in northeastern Brazil. Like other stingless bees, this species plays an important ecological role as a pollinator. The aim of the present study was to investigate the genetic structure and evolutionary history of *P*. *seridoensis* across its current geographic range. Workers from 84 nests from 17 localities were analyzed for COI and Cytb genic regions. The population structure tests (Bayesian phylogenetic inference, AMOVA and haplotype network) consistently characterized two haplogroups (northwestern and eastern), with little gene flow between them, generating a high differentiation between them as well as among the populations within each haplogroup. The Mantel test revealed no isolation by distance. No evidence of a potential geographic barrier in the present that could explain the diversification between the *P*. *seridoensis* haplogroups was found. However, Pleistocene climatic changes may explain this differentiation, since the initial time for the *P*. *seridoensis* lineages diversification took place during the mid-Pleistocene, specifically the interglacial period, when the biota is presumed to have been more associated with dry conditions and had more restricted, fragmented geographical distribution. This event may have driven diversification by isolating the two haplogroups. Otherwise, the climatic changes in the late Pleistocene must not have drastically affected the population dynamics of *P*. *seridoensis*, since the Bayesian Skyline Plot did not reveal any substantial fluctuation in effective population size in either haplogroup. Considering its importance and the fact that it is an endemic bee from a very threatened Neotropical dry forest, the results herein could be useful to the development of conservation strategies for *P*. *seridoensis*.

## Introduction

Social bees from the tribe Meliponini, known as stingless bees, are distributed in tropical and southern subtropical areas throughout the world, with greater diversity in Neotropical and Indo-Malayan regions [1,2]. So far, a total of 641 names for stingless bees are listed for the Neotropical region, 417 of which are considered valid [2]. Recent data indicate 244 valid species and another 89 undescribed forms [3] occurring in Brazil alone. Stingless bees play an important ecological role as pollinators of many wild plant species and are considered key elements for the maintenance and conservation of natural ecosystems as well as economically important agricultural systems [4]. However, recent studies have shown the decline of these pollinators and the consequent loss of pollination services in many areas of the world due mainly to human activities [5,6,7,8]. Despite the diversity, importance and threats, little is known regarding the population genetics and phylogeography of these bees.

The stingless bee *Partamona seridoensis* [9] occupies xeric regions, such as the *Caatinga* (phytogeographic domain), a Neotropical dry forest in the northeastern region of Brazil. This species nests only in active arboreal termite nests, mainly those of *Constrictotermes cyphergaster*. (Termitidae, Nasutitermitinae) and *Microcerotermes* spp [9,10,11]. *Partamona seridoensis* has been poorly studied and only one report is found in the literature on its population genetics, in which two populations were analyzed using allozymic and microsatellite markers [12]. There have been no studies on its conservation status, although this bee occupies the *Caatinga*, which is a threatened region. A recent study involving *Partamona rustica* has suggested the importance of concentrating efforts on the conservation of endemic stingless bees from Brazilian dry forests, due to bee hunters and deforestation, which have threatened these populations [13].

The *Caatinga* covers nearly 85,000 Km^2^ and is a highly heterogeneous phytogeographical domain, being the second largest center of endemism of xeric environments in South America [14,15]. Studies on animals and plants of the *Caatinga* have shown that this environment harbors high levels of species richness and specific interactions between insects and plants [13,14,16,17]. However, the *Caatinga* is one of the least protected areas by conservation policies in Brazil, with only 5.9% of its area included within conservation units [18], and one of the least studied areas of the Neotropical dry forest diagonal, constituted by the *Chaco*, *Cerrado* and *Caatinga* phytogeographic domains [14]. Moreover, deforestation combined with the semi-arid climate of the *Caatinga* has led to a process of desertification in many areas, making this phytogeographical domain severely threatened by climate changes [19].

Many studies have shown the effects of Pleistocene climatic events on the population structure and demography of bees around the world [20,21,22]. However, these studies are scarce for endemic bees in xeric habitats, such as the Neotropical dry forest diagonal [23], especially with regard to stingless bee populations of the *Caatinga*. A recent study analyzed the genetic structure and evolutionary processes involved in the current genetic variation of *Melipona subnitida*, an endemic species in northeastern Brazil, and suggested that the paleoclimatic changes of the Pleistocene could have played an important role in the diversification of this species [24]. Another recent study reconstructed the evolutionary history of *Partamona rustica*, an endemic stingless bee from the Neotropical dry forest in Brazil, and identified two groups of populations with no evidence of substantial changes in effective population size during the late Pleistocene [23].

Understanding the evolutionary history of a species and its genetic structure is essential to the development of effective conservation strategies [23,24], especially for species of the threatened *Caatinga*. Given the potentially serious consequences of inbreeding in stingless bees due the complementary sex determination mechanism [25,26], it is essential to understand the genetic population structure of these organisms to enable the implementation of management strategies and conservation policies. Thus, the aim of the present study was to investigate the genetic structure and evolutionary history of *P*. *seridoensis* across its current geographic range.

## Materials and methods

### Sampling

**Fig 1:**
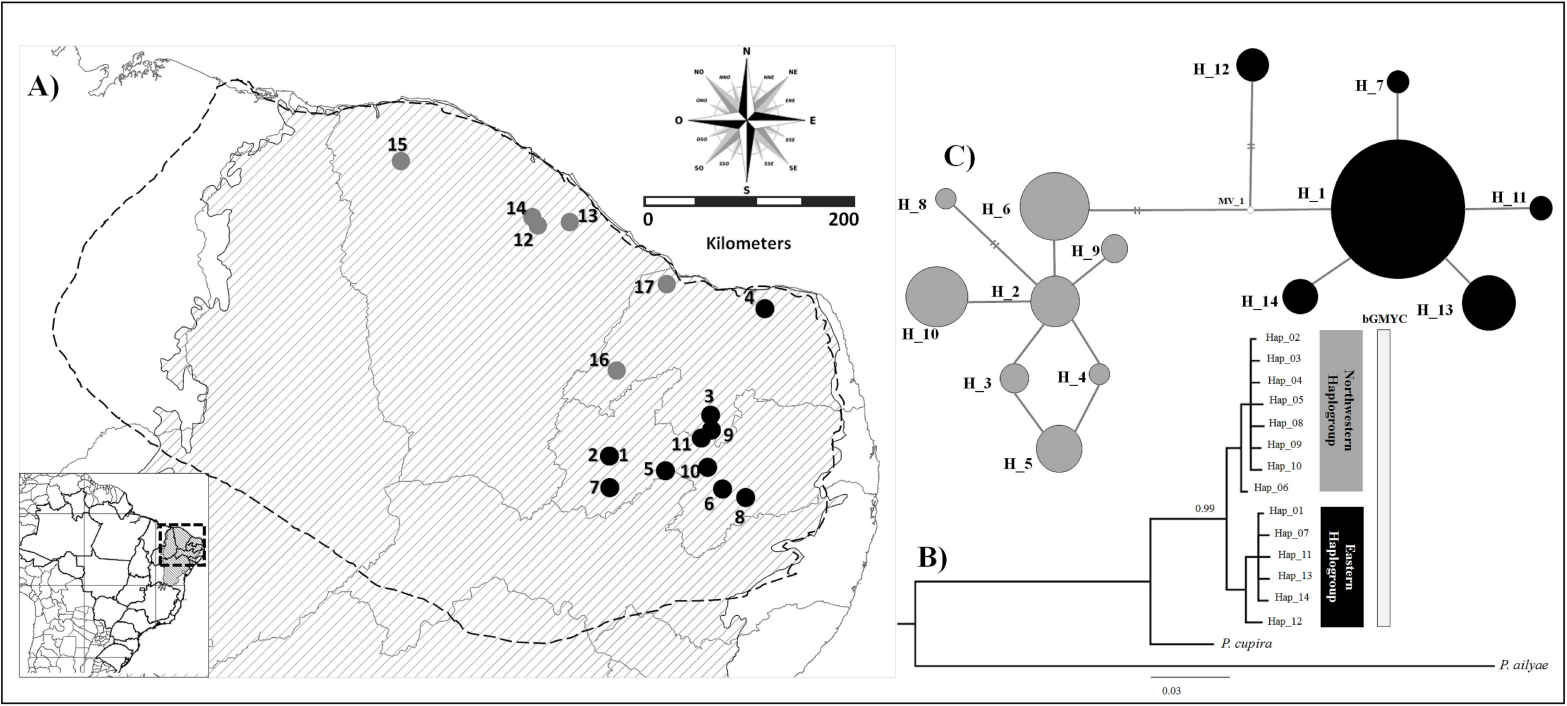
**Geographical distribution of *P*. *seridoensis* populations (A). Samples are indicated by circle and number and listed according to Table 1. Bayesian inference using concatenated COI and Cytb mitochondrial gene regions in *P*. *seridoensis* and outgroups (B). Median-joining haplotype network in *P*. *seridoensis* (C).** *Caatinga* area is represented by downward diagonal lines and the dotted line represents the geographical distribution of *P*. *seridoensis* [9] in **A**. Bayesian implementations of the Generalized Mixed Yule Coalescent model (bGMYC) for *P*. *seridoensis*, shown in B. Two lines represent two mutation steps between haplotypes and no line between haplotypes represents one mutation step in C. Gray color represents northwestern haplogroup and black color represents eastern haplogroup (in all figures).

Adult workers were collected from *P*. *seridoensis* nests from March 2008 to August 2014. Through active searches, 84 nests were sampled from 17 localities in the northeastern region of Brazil, with an average of five nests per locality (Table 1 and Fig 1A). The sampled localities were chosen through prior knowledge of occurrence records available on online database and others studies as well as through our active searches. In each locality where nests of *P*. *seridoensis* were sampled, we look for nests distant from each other at least 300 meters to avoid high kinship between them. All specimens were preserved in ethanol prior to the molecular analyses and vouchers were deposited in the Camargo Entomological Collection at the Universidade de São Paulo (FFCLRP—USP) and in the Laboratório de Genética Evolutiva de Himenópteros at the Universidade Federal de São Carlos (LGEH–UFSCar). All necessary research permits for fieldwork and collection of samples were issued by the Brazilian Institute for Biodiversity Conservation (Instituto Chico Mendes de Conservação da Biodiversidade—ICMBio) recorded by SISBio (permit number 31750). Field studies did not involve endangered or protected species.

**Table 1:** Geographical origin (with coordinates) of the *P*. *seridoensis* nests. N = number of colonies; H = haplotypes found in each locality.

	Localities	Code	Latitude	Longitude	N	H
1	Almas-PB	ALM	-7.4730	-36.904	10	H1
2	Moreiras-PB	MOR	-7.3933	-36.414	10	H1
3	Jardim do Seridó-RN	JSE	-6.5976	-36.81	1	H7
4	Jandaíra-RN	JAN	-5.3508	-36.487	10	H1
5	Matureia-PB	MAT	-7.2623	-37.351	5	H1
6	Serra Branca-PB	SBR	-7.4808	-36.666	3	H1
7	Nova Olinda-PB	NOL	-7.4645	-38.017	1	H1
8	São Domingos do Cariri-PB	SDC	-7.5824	-36.391	1	H11
9	São José do Sabugi-PB	SJS	-6.776	-36.799	2	H12
10	Taperoá-PB	TAP	-7.2216	-36.846	2	H13
11	Santa Luzia-PB	SLU	-6.8718	-36.921	6	H14
12	Baturité-CE	BAT	-4.3292	-38.881	6	H2
13	Chorozinho-CE	CHO	-4.2891	-38.499	8	H3, H4, H5
14	Guaramiranga-CE	GUA	-4.23	-38.948	10	H6
15	Alcântaras-CE	ALC	-3.5588	-40.5210	1	H8
16	Martins-RN	MAR	-6.0635	-37.937	1	H9
17	Mossoró-RN	MOS	-5.1870	-37.624	7	H10
				**Total**	**84**	-

### Amplification and sequencing

Total DNA was extracted from one worker per colony using a phenol-chloroform protocol [27]. Fragments of the mitochondrial genes cytochrome c oxidase I (COI) [28] and cytochrome *b* (Cytb) [29], commonly used for this type of study, were amplified. All polymerase chain reactions (PCR) contained template DNA (50 ng), 1X of Taq buffer (Invitrogen, USA), 250 μM of each dNTP, l.0 μM of each primer, 2.5 mM of MgCl_2_ and 1 U of Platinum Taq DNA polymerase (Invitrogen, USA) in a final volume of 25 μL. The PCR conditions for all gene regions were as follows: initial denaturation at 94°C for 5 minutes, followed by 40 cycles of denaturation at 94°C for 30 s, annealing at 50°C and extension at 72°C for 1 min, and completed with an additional extension step at 72°C for 10 min. The PCR products were electrophoresed in agarose gel stained with GelRed^TM^. The PCR product was purified using the Illustra ExoProStar 1-Step Kit (GE Healthcare, Buckinghamshire, UK). The mitochondrial genes were sequenced in both directions with the same amplification primers using the BigDye v 3.0 Dye Terminator Cycle Sequencing Kit (Applied Biosystems, Inc., Carlsbad, CA, USA) in an automated sequencer ABI 3730 XL (Applied Biosystems).

### Genetic diversity

The electropherograms were edited using the Codon Code v3.7.1 program (CodonCode, Dedham, Massachusetts, USA). Sequences were aligned using the Clustal W algorithm with the aid of the BioEdit 7.0.9.0 program [30]. All alignments were visually inspected and corrected. All haplotype sequences were deposited in the GenBank (accession numbers KX839192-KX839206). The number of variable sites (S), number of haplotypes (h), haplotype diversity (Hd) and nucleotide diversity (π) considering the concatenated gene regions were estimated using the DnaSP v5.10.01 program [31].

### Phylogeographic structure tests

Bayesian inference was used to estimate the phylogeny of *P*. *seridoensis* lineages based on the concatenated mitochondrial haplotypes. The model with the best fit was selected with the aid of the MrModeltest v2.2 program [32] based on the Akaike information criterion (AIC) for two partitions (COI and Cytb). Two independent Bayesian runs of 20 million generations were performed, each with four Markov Chain Monte Carlo (MCMC) simulations. The first two thousand generations were discarded as burn-in, after which trees were sampled every 1000 generations. This analysis was performed using the MrBayes 3.1.2 program [33]. Chain convergence was checked using likelihood plots for each run with the aid of the Tracer 1.5 software [34] and results with ESS values > 200 were accepted. The consensus species tree for each inference was visualized and edited using the FigTree 1.4 software (tree.bio.ed.ac.uk/software/figtree/). For this analysis, *Partamona cupira* and *Partamona ailyae* were used as the outgroup, since these species are part of the same group as *P*. *seridoensis*.

To check if the haplogroups observed are not cryptic species and assess uncertainty in phylogenetic tree estimation and model parameters, a Bayesian implementation of the generalized mixed Yule coalescent model (bGMYC) was used, which integrates over these potential sources of error via MCMC simulation [35]. The R package ‘bGMYC’ was used to calculate marginal posterior probabilities of species limits from the posterior distribution of ultrametric trees reconstructed by BEAST 1.8.0. A post-burn-in sample of 100 trees resampled from that posterior was used to calculate the posterior distribution of the bGMYC model. The bGMYC analysis was run for 100,000 generations, with a burn-in of 90,000 generations and a thinning interval of 100 samples.

Haplotype networks were constructed employing a median-joining network [36] in the NETWORK program (www.fluxus-engineering.com/sharenet.htm). Analysis of molecular variance (AMOVA) was performed to estimate the degree of population genetic differentiation among populations using three hierarchical levels. The Φ_ST_ and Φ_CT_ indexes were also estimated. These analyses were implemented using the ARLEQUIN 3.11 program [37] and significance was determined with 1000 permutations. A Mantel test was carried out using the program IBDWS v.3.23 [38] to test the hypothesis of isolation by distance, considering each haplogroup separately and all populations together. Significance of this analysis was determined with 1000 permutations. All these analyses were implemented using the concatenated gene regions.

### Population divergence time

A Bayesian approach implemented in BEAST 1.8.0 program [39] was used to estimate the time since the most recent common ancestor (TMRCA) of all samples and for each recovered lineage, based on the concatenated regions. Two independent runs were implemented for each estimate with the following parameters: coalescent constant size as a tree prior, UPGMA as an initial tree, a strict clock, 100 million generations, parameter sampling every 5000 generations and 10% of the MCMC simulations as burn-in. As there is currently no measure of mitochondrial mutation rate for corbiculate bees, to obtain the absolute times, a COI substitution rate of 1.9% per lineage per million years per generation (one year) (under a normal distributed prior) calibrated for other hymenopterans [40] was adopted. The substitution models for the northwestern and eastern haplogroups (see Results) were TrN and HKI+I, respectively, which were selected using the jModeltest v.2.1.5 program [41] and based on the AIC. Convergence between runs and analysis performance were checked using Tracer 1.5. Results with ESS values > 200 were accepted. Independent runs of each TMRCA estimate were combined in LogCombiner [39].

### Demographic inference

A Bayesian Skyline Plot (BSP) reconstruction was implemented for possible groups identified in the phylogenetic reconstruction to evaluate population size dynamics over time using the BEAST 1.8.0 program [39]. All the concatenated sequences for each group were used. As no selection was observed for each gene, as well as for their respective codon positions (data not shown), we used all codon positions for each gene on this analysis. The evolutionary model, clock and genes regions were the same as those used in TMRCA inference. The following parameters were employed: 20 million generations for each haplogroup, parameter sampling every 1000 generations of the MCMC analysis and a 10% burn-in period. Convergence between runs and the performance of the analysis were checked using Tracer 1.5 and the plots were constructed with the aid of the same program [34].

### Gene flow and migration

We evaluated the gene flow between haplogroups by the structure tests using Migrate-n v3.6 [42]. The migration parameters were estimated using concatenated gene regions. Migrate-n v3.6 estimates migration rate divided by the mutation rate *M* (*m*/μ). Four migration models were tested and compared by the bayes factor: (1) a full model with two population sizes and two migration rates; (2) a model with two population sizes and one migration rate (gene flow into the northwestern population); (3) a model with two population sizes and one migration rate (gene flow into the eastern population); (4) a model considering eastern and northwestern as a panmictic population. We used a Bayesian approach and thermodynamic integration of four chains with a static heating swap scheme (temperatures: 1.0, 1.5, 3.0, 10^6^), with two independent runs, sampling at every 600th step for a total of 600,000 of recorded steps and a burn-in of 150,000 steps.

## Results

### Genetic diversity

A fragment of 1,015 base pairs (Cytb– 433 bp; COI– 582 bp) was obtained for the concatenated genes of *P*. *seridoensis* and no indels occurred in these amplicons. We have no ambiguous bases or missing data in our dataset. Sixteen variable sites and 14 haplotypes were identified. The species exhibited low nucleotide diversity (π = 0.00266 ± 0.00019) and high haplotype diversity (Hd = 0.756 ± 0.044) (Table 2).

**Table 2:** Genetic diversity of *P*. *seridoensis*. Number of samples (N), number of haplotypes (h), variables sites (S), nucleotide diversity (π) and haplotype diversity (Hd) for concatenated mitochondrial genes COI and Cytb in each haplogroup and all populations.

Haplogroups	N	h	S	π	Hd
Eastern	51	6	7	0.00059 (±0.00017)	0.405 (±0.082)
Northwestern	33	8	7	0.00159 (±0.00015)	0.826 (±0.034)
***All populations***	84	14	16	0.00266 (±0.00019)	0.756(±0.044)

### Phylogeographic structure

Based on the AIC, the evolutionary model selected for the Bayesian Inference, including the outgroup, was GTR + I. Fig 1B shows the topology based on this inference. Two haplogroups with a high value of branching support were identified. The first, eastern haplogroup, comprised samples from 11 locations (1 to 11 in Table 1), in which six haplotypes were identified. In this haplogroup, haplotype H1 was shared among six populations and all the other haplotypes were privative of the localities (black haplotypes in Fig 1C). The second, northwestern haplogroup, comprised samples from six locations (12 to 17 in Table 1), which exhibited eight haplotypes that were privative of the locations (grey haplotypes in Fig 1C). The bGMYC shows both haplogroups as a single evolutionary entity, with poor posterior probability values (PP< 0.9). AMOVA indicated high differentiation between haplogroups and among populations (Φ_CT_ = 0.740, *p* < 0.0001; Φ_ST_ = 0.984; *p* < 0.0001, respectively).

The Mantel test demonstrated no correlation between genetic and geographic distances considering all populations (r = 0.171; *p* = 0.097) and populations in each haplogroup separately (r_eastern_ = -0.222, *p* = 0.812; r_northeastern_ = 0.245, *p* = 0.2468), indicating no isolation by distance among populations.

### Divergence time and demographic inferences

**Fig 2:**
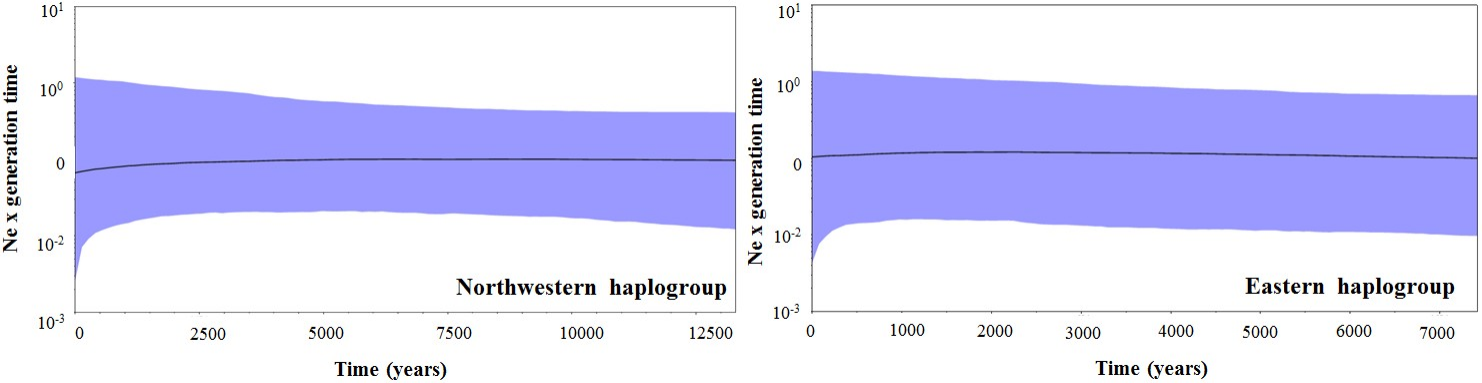
Coalescent Bayesian Skyline Plot (BSP) used to infer demographic history of *P*. *seridoensis* population groups. Black horizontal line shows median BSP estimate and blue area shows upper and lower 95% highest posterior density limits.

The coalescent-based analysis considering all *P*. *seridoensis* populations indicated a TMRCA of 163 kya (highest posterior density [HPD 95%:]: 87.8–257.6 kya), suggesting that this could be the initial diversification time for the two *P*. *seridoensis* lineages analyzed herein. The TMRCA for each group indicated a recent age for these two lineages, with coalescence of northwestern and eastern groups respectively occurring 84.5 kya (HPD 95%: 35.7–149.6 kya) and 106.9 kya (HPD 95%: 49.3–180.5 kya). The BSP did not reveal any substantial fluctuations in the effective population size (Ne) in either haplogroup (Fig 2).

### Migration rates

We detected little gene flow between the eastern and northwestern populations in all models. Migration from the eastern to the northwestern populations was the most probable model (0.72) of gene flow (Table 3), with the number of migrants per generation (Ne*m*_eastern_) of 0.63 (95% credibility interval: 0–2.87 migrants). All these parameters showed posterior probability curves with unique peaks and effective sample sizes (ESS) higher than 1,000, indicating convergence in the run.

**Table 3:** Bayes factors and log marginal likelihoods of the four evaluated migration models between northwestern and eastern haplogroups of *P*. *seridoensis*.

	Bezier lmL	LBF(Bezier)	Choice(Bezier)	Model probability
M_eastern>northwestern_	-1446.0564	0.0000	1	0.72582
M_northwestern>eastern_	-1447.0697	-2.0266	2	0.2634
Full	-1450.2751	-8.4374	3	0.0106
Panmictic	-1458.5843	-25.0558	4	2.63042E-06

## Discussion

The population structure tests (phylogenetic inference, AMOVA and haplotype network) consistently characterized two haplogroups (northwestern and eastern). The results revealed a high number of privative haplotypes and a high level of differentiation between haplogroups as well as among all populations, with little gene flow mainly from the eastern to the northwestern haplogroup when the population genetic structure is assessed by mitochondrial genes. Moreover, the bGMYC analysis recovered a single evolutionary entity, suggesting that these haplogroups are not different species or even cryptic species. Previous study involving allozymic and microsatellite loci found a low population differentiation between two geographically close *P*. *seridoensis* populations in the state of Paraíba (Brazil) [12], suggesting restrictions to gene flow between the populations as the present study. Some biological traits of stingless bees limit dispersal. New queens of species of *Partamona* are philopatric, remaining in the place of origin and not flying more than 300 meters from the maternal nest during the swarming process [43,44,45]. Moreover, *P*. *seridoensis* builds its nests on specific substrates, nesting in arboreal termite nests [9,10,11]. Since stingless bees exhibit limited dispersal capacity, relatively weak geographical barriers may effectively restrict gene flow [46].

There is no evidence of potential geographic barriers (such as mountains, large rivers or valleys) in the present that could explain the diversification found between the two haplogroups of *P*. *seridoensis*. Moreover, no correlation was found between genetic and geographical distances, suggesting no isolation by distance among the populations analyzed. However, the differentiation that resulted in the two haplogroups may be explained by climatic changes in the mid-Pleistocene, since the initial diversification time for these lineages was estimated at approximately 163 kya, specifically in the interglacial period [47], a time when the biota is presumed to have been more associated with dry conditions and had more restricted and fragmented geographical distribution [24]. This event may have driven the diversification process, isolating northwestern and eastern haplogroups. Furthermore, like other stingless bees, *P*. *seridoensis* depends on plant resources, such as pollen, nectar and resins, as well the existence of suitable sites for nesting, such as termite nests. Such resources may also have had restricted geographical distribution due to climatic changes. These aspects and the presumed limited dispersal capacity of this stingless bee lend support to the hypothesis that mid-Pleistocene climatic changes may have driven the diversification of *P*. *seridoensis* into at least two haplogroups.

Similar results were observed to populations of *Melipona subnitida*, a stingless bee with a similar distribution area in the *Caatinga* [24]. The authors characterized two evolutionary lineages of the species, which also diverged in the mid-Pleistocene, and suggest that paleoclimatic changes in this period could have played an important role in this diversification. Other phylogeographic studies involving species associated with xeric vegetation in interglacial periods report data that are in agreement with the present findings [23,48,49,50]. Some researchers have also shown that climate oscillations in the Pleistocene may have promoted changes in the range of Neotropical forests in response to more warm and humid (interglacial periods) or more cold and dry (glacial periods) climatic conditions [51,52,53]. Moreover, climatic changes may have also influenced biogeographic patterns, genetic diversity, historical demography and species richness in the Neotropical biota [54,55] as well as the biota of other regions around the world [20,21,56].

Studies have suggested that forests expanded into the *Caatinga*, at least locally, during the wettest periods [57,58]. Population expansion for two evolutionary lineages of *M*. *subnitida* in the *Caatinga* during the Pleistocene period was reported [24]. However, the late Pleistocene climatic changes do not seem to have had a substantial influence on the demographic patterns of the *P*. *seridoensis* groups, since the BSP did not reveal any substantial fluctuations in the effective size of the populations in either haplogroup analyzed herein (Fig 2). As the two *P*. *seridoensis* haplogroups have a very recent origin, diverging after the Last Interglacial Maximum (approximately 120 kya), oscillations occurred in the late Pleistocene must not have drastically affected the population dynamics. A putative refuge area for Seasonally Dry Tropical Forests in the *Caatinga* between the Last Glacial Maximum and the present, which covers most of the current distribution area [59] was recently proposed. Such an area may have served as refuge for the species during this period, which allowed the demographic stability of the two lineages. This finding is in agreement with another study, which also found that climatic changes of the late Pleistocene did not seem to have a remarkable influence on the demographic patterns of *P*. *rustica* populations in Brazilian dry forests [23].

Unfortunately, there are no substitution rates yet available for the mtDNA (such as COI or Cytb genes) of bees. Therefore, recent studies involving bees [22,23,24] published in important journals have also used calibration described for other insects to estimate divergence time as well as historical demography. Studies have shown that the use of a “universal” mutation rate may overestimate the timing of population-level events [60,61,62]. It should be noted that the rate used in the present study agrees with others calibration rates estimated for the COI of other insects, such as 1.28 for beetles [63], 1.45 for ants [64] and 2.0 for *Drosophila* [65], as well as the COI and Cytb concatenated regions, such as 1.5–2.0 for Orthoptera [66] 0.8–1.3 for Diptera [67] and 2.1 for Coleoptera [68]. Therefore, the rate used in the present study does not differ much from those estimated for others insects and should not markedly compromise the results obtained herein.

The population differentiation of *P*. *seridoensis* found with mitochondrial genes is expected to be lower when assessed using nuclear genes, as observed in a previous study involving the population genetics of *P*. *seridoensis* using nuclear markers [12]. Like most Hymenopterans, stingless bees have a single multiallele locus that controls sex determination [26,69,70], in which hemizygous individuals are males, whereas diploid individuals that are heterozygous will develop into females and those homozygous at this sex locus will be diploid males [71]. Thus, this model predicts that inbreeding should produce diploids males that are highly harmful to colonies and populations. Due to the philopatric behavior of females, gene flow among populations must be accomplished by males, leading to asymmetrical sex dispersal, as also observed in other stingless bees [12,23,72,] and even bees from the tribe Euglossini in Neotropical regions [22,73,74,]. Hence, one would expect lower differentiation among populations based on nuclear genes due to the dispersal performed by males in these bee populations [23,72].

Recent study stressed the need for further, more detailed investigations of dry environments of the Neotropical region, since few phylogeographical studies have considered the biota of this area [23]. The present results contribute to a better understanding of evolutionary processes occurring in the *Caatinga* during the Pleistocene, as we have shown that the lineages of *P*. *seridoensis* diversified during the mid-Pleistocene likely due to climatic changes, resulting in two haplogroups. The conservation of different evolutionary lineages is important to preserving better adapted genotypes for greater survival during drought periods in the *Caatinga* [24]. Moreover, for species with low population gene flow and, consequently, high differentiation, as observed in the present study, it is important to preserve local populations in order to maintain the species throughout its range, which is an important source of information for planning conservation strategies [75,76]. Recent studies have shown that bee hunters, intense deforestation in Caatinga areas and livestock farming represent serious threat to stingless bees [13,23,77]. Studies involving organisms from dry forests as Caatinga are important since it has been excluded from discussions on conservation [78]. Given its importance as a pollinator and the fact that it is an endemic bee from a threatened dry forest, the present findings can be useful to the development of conservation strategies aimed at *P*. *seridoensis* populations, especially its two evolutionary lineages. Conservation policies directed at preserving pollinating species may be an effective mechanism for preserving the biodiversity of Neotropical biomes, which is an urgent task that must be pursued.
